# Evidence for a complex formation between CYP2J5 and mEH in living cells by FRET analysis of membrane protein interaction in the endoplasmic reticulum (FAMPIR)

**DOI:** 10.1007/s00204-017-2072-0

**Published:** 2017-10-13

**Authors:** Anette Carolina Orjuela Leon, Anne Marwosky, Michael Arand

**Affiliations:** Institute of Pharmacology and Toxicology, University of Zurich, Winterthurerstrasse 190, 8057 Zurich, Switzerland

**Keywords:** Enzymatic detoxification, Lipid signaling, Protein interaction analysis

## Abstract

The potential complex formation between microsomal epoxide hydrolase (mEH) and cytochrome P450-dependent monooxygenase (CYP) has been a subject of research for many decades. Such an association would enable efficient substrate channeling between CYP and mEH and as such represent an attractive strategy to prevent deleterious accumulation of harmful metabolic by-products such as CYP-generated epoxide intermediates. However, such complex formation is experimentally difficult to prove, because CYP and mEH are membrane-bound proteins that are prone to unspecific aggregation after solubilization. Here, we report the development of a FRET-based procedure to analyze the mEH–CYP interaction in living cells by fluorescence-activated cell sorting. With this non-invasive procedure, we demonstrate that CYP2J5 and mEH associate in the endoplasmic reticulum of recombinant HEK293 cells to the same extent as do CYP2J5 and its indispensible redox partner cytochrome P450 reductase. This presents final proof for a very close proximity of CYP and mEH in the endoplasmic reticulum, compatible with and indicative of their physical interaction. In addition, we provide with FAMPIR a robust and easy-to-implement general method for analyzing the interaction of ER membrane-resident proteins that share a type I topology.

**Electronic supplementary material:**

The online version of this article (doi:10.1007/s00204-017-2072-0) contains supplementary material, which is available to authorized users.

## Introduction

Among the many oxygenation reactions catalyzed by cytochrome P450-dependent monooxygenases (CYP), epoxygenation is a particularly important one (Guengerich 2003; Spector 2009). The resulting epoxides vary substantially in their stability/reactivity. While some epoxides, such as the 8,9-*exo*-epoxide of Aflatoxin B_1_, are highly electrophilically reactive and represent a major threat, due to their reactivity with DNA and proteins, other epoxides are very stable and poorly reactive (Guengerich 2003). This allows the body to use compounds of the latter type, for instance the arachidonic acid-derived epoxyeicosatrienoic acids (EETs), as signaling molecules (Spector 2009). Both, the DNA-reactive as well as the signaling epoxides can be readily hydrolyzed by epoxide hydrolases (EH), which results in a substantial modification of their biologic activity (Marowsky et al. 2010).

**Fig. 1:**
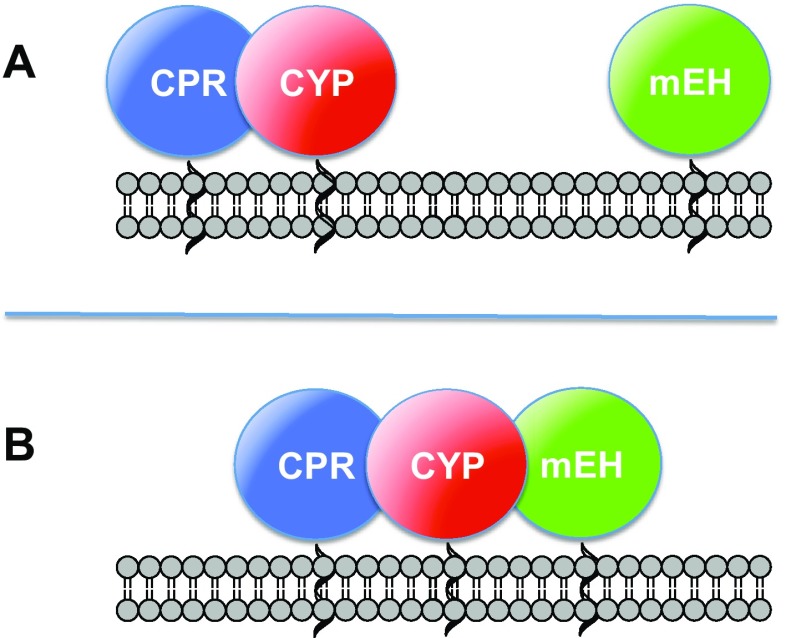
Different options for the association behavior of CYP and mEH in the endoplasmic reticulum: **a** CYP and mEH remain essentially unassociated, or **b** CYP and mEH form a complex. The association of CYP and CPR is well established and thus can be used as a positive control. *mEH* microsomal epoxide hydrolase, *CPR* cytochrome P450 reductase, *CYP* cytochtome P450-dependant monooxygenase

The two major mammalian EHs, the soluble (sEH) and the microsomal epoxide hydrolase (mEH), differ in their subcellular localization; sEH is mainly a cytoplasmic enzyme with a moderate fraction localized in peroxisomes of certain cell types (Enayetallah et al. 2006). In contrast, mEH, like the majority of CYPs, is an endoplasmic reticulum (ER)-resident protein. With respect to their membrane topology, CYP and mEH are type-I proteins (Black 1992; Friedberg et al. 1996). Their short N-termini that precede their single transmembrane helix face the ER lumen while their catalytic domains are sitting on the cytoplasmatic surface of the ER. CYP is invariably associated with cytochrome P450 reductase (CPR), the mandatory electron donor for the first step in the CYP catalytic cycle (Lu et al. 1969). However, whether CYP and mEH, when expressed within the same cell, also form a complex (Fig. 1) to facilitate the production of the final metabolites, thus reducing the accumulation of epoxide intermediates, is still a matter of debate.

Such complex formation has already been postulated some decades ago (Oesch and Daly 1972) and several different attempts have been undertaken to prove its existence (Etter et al. 1991; Fujiwara and Itoh 2014; Guengerich and Davidson 1982; Oesch and Daly 1972; Taura Ki et al. 2002; Taura et al. 2000). However, methodologic limitations of the strategies applied to the problem still leave some doubt with respect to the validity of the resulting findings, as will be explained in the Discussion below.

**Fig. 2:**
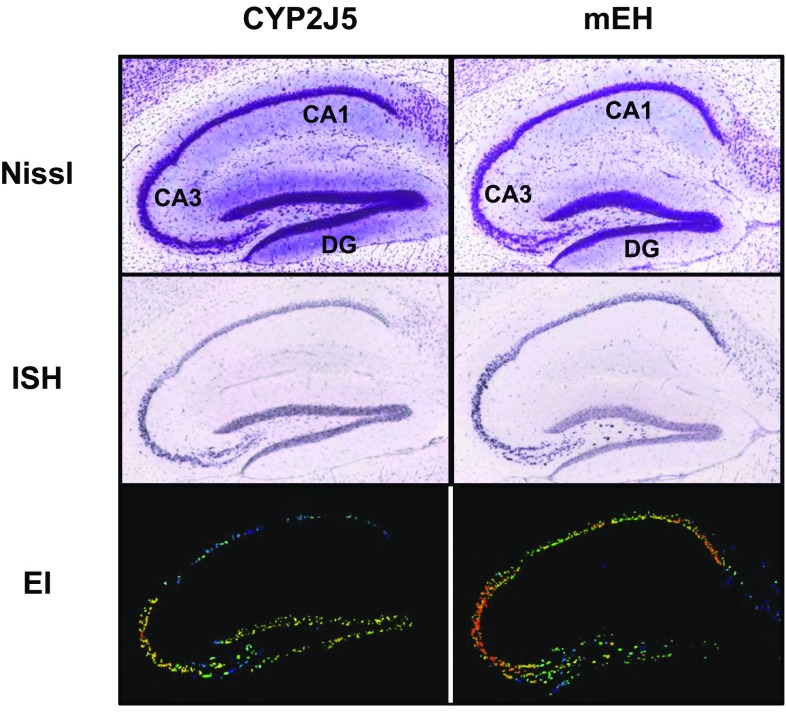
CYP2J5 and mEH expression in the adult mouse hippocampus. In situ hybridization analysis of CYP2J5 and mEH mRNA expression (middle row), compared with (upper row) and normalized to (lower row) a general nucleic acid staining in the adult mouse hippocampus. Pictures represent sagittal sections taken from the Allen brain atlas (link: http://mouse.brain-map.org), specifically experiments 513,229 (CYP2J5) and 68,498,247 (mEH) (Lein et al. 2007). Note that the expression levels for both mRNAs are highest in the CA3 region of the hippocampus and indicate strong expression in the principal neurons of this region. *CA1* cornu ammonis area 1, *CA3* cornu ammonis area 3, *DG* dentate gyrus, *EI* expression index (ISH signal intensity normalized to Nissl staining); *ISH* in situ hybridization with specific antisense probes, *Nissl* Nissl staining for nucleic acids

In the current study, we have developed a FRET-based approach to demonstrate close proximity of ER-residing type I membrane proteins, including CYP and mEH, which we call FAMPIR, for FRET analysis of membrane protein interactions in the endoplasmic reticulum. To overcome the sensitivity problems associated with the limited expression yields of ER-resident proteins, we used FACS analysis of the FRET signal to obtain statistically robust results. As a first potentially relevant member of the CYP family that might interact with mEH we chose CYP2J5, because (i) it is coexpressed with mEH in specific CNS cell populations, namely the principal neurons of the hippocampus (Fig. 2), and (ii) it produces 11,12-epoxyeicosatrienoic acid (Ma et al. 1999), a lipid signaling molecule efficiently hydrolyzed by mEH (Marowsky et al. 2009).

Due to the essentially non-invasive nature of FAMPIR, we were able to unequivocally demonstrate the direct interaction of CYP2J5 and mEH in recombinant HEK 293 cells. Furthermore, we present with FAMPIR a fast and robust method that is generally applicable to the interaction analysis of type-I ER-resident proteins.

## Methods

### Vector construction

A detailed description and graphical representation (Fig. S1) of the expression vector construction is given in the Supplementary Information.

### Transfection of HEK293 cells

HEK293 were cultivated in DMEM medium (Gibco^®^, Thermo Scientific, Waltham, MA, USA) supplemented with 5% FCS and 2 mM l-glutamine at 37 °C under 5% CO_2_. When reaching 80% confluency they were split using 0.25% Trypsin/EDTA (Gibco^®^, Thermo Scientific, Waltham, MA, USA) for the detachment of the cells. Cells were seeded on sterile, nitric acid-treated glass coverslips placed in 12 well (18 mm) dishes for subsequent microscopic analyses, or in 6 cm dishes for subsequent FACS analyses. Transient transfection was carried out using Jet-PEI™ reagent (Polyplus-Transfection, USA), following the manufacturers protocol.

FRET analyses by confocal microscopy were performed on an LSM710 confocal microscope (Carl Zeiss AG, Oberkochen, Germany) equipped with an EC Plan-Neofluar 40×/1.30 Oil DIC M27 objective. The CFP channel was excited with the 458 nm laser line and emission was recorded at 470–485 nm. The YFP channel used the 514 nm laser line and emission was recorded at 521–540 nm. Forty-eight hours after transfection, cells on coverslips expressing the fluorescent chimeras were washed once with PBS, mounted in a live imaging chamber and covered with 1 ml HEPES-based imaging medium (1× Live Cell Imaging Solution, Molecular Probes, USA). Cells were analyzed at 37 °C for a maximum of 45 min. After CFP and YFP colocalization was assessed, images were taken using the multichannel acquisition mode. For the analysis of acceptor photobleaching, images were first taken from an appropriate field of cells in the CFP and YFP channels. Then, YFP photobleaching was applied with 50 iterations for 0.3 ms using the 514 nm laser at 100% intensity. Thereafter, images were taken again. The degree of FRET was calculated using the Image J analysis software by estimating the degree of CFP dequenching due to the destruction of the YFP in a given region of interest.

### FRET analysis by FACS

This procedure was adapted from Banning et al. (2010), Thyrock et al. (2010), with some minor modifications. Forty-eight hours after transfection, cells were trypsinized and resuspended in 500 µl of DMEM without phenol red, containing 5% FCS. Cell density was approximately 2 × 10^6^ cells/ml. FRET analyses were carried out on an LSR II Fortessa (BD Biosciences, Franklin Lakes, NJ, USA), using the sensitized emission method with the following filter settings: YFP channel (488 nm, 530/30 filter, 505 LP), CFP channel (405 nm, 450/50 filter) and the FRET channel (405 nm 525/50 filter, 505 LP). During analysis, samples were first gated according to forward scatter (FSC) and side scatter (SSC) characteristics to ensure that only single cells were analyzed. A total of 5 × 10^4^ cells per sample meeting these criteria were included. Using cells expressing only CFP or YFP chimeras, background or false FRET signals due to bleed-through were efficiently corrected for with the built-in compensation procedure provided by the BD FACSDiva™ software. The subpopulation displaying significant YFP and CFP expression was selected and analyzed for FRET in a CFP vs FRET plot. For each session of recordings, the selection gate P2 defining the FRET-positive events was individually adjusted, based on the respective measurements obtained with cells expressing only either CFP or YFP chimera to compensate for potential day-to-day variations in any step of the analytical procedure.

### Proximity ligation assay (PLA)

For the isolation of primary hippocampal neurons, hippocampi were dissected from the brains of newborn C57Bl6/J mice (P0-2), cut into small pieces, rinsed with ice cold PBG [glucose, 10 mM, and BSA, 1 mg/ml, in phosphate buffered saline (PBS)], and subsequently digested in PBG containing 0.5 mg papain and 1 mg DNAse per ml at 37 °C in a volume of 2.5 ml per animal. After 10 min, the supernatant was aspirated and the remaining tissue was washed twice with DMEM. Finally, cells were dissociated in 1.5 ml DMEM containing 10% FCS and 2 mM l-glutamine using a fire-polished Pasteur pipette. This suspension was seeded on polylysine-precoated coverslips and subsequently incubated in 2 ml mouse hippocampal medium [MEM/B27/glucose (Gibco^®^, Themo Scientific Fischer, MA, USA)]. After 48 h, 20 µl of a solution of 3.5 mg uridine and 1.5 mg 5-fluoro desoxyuridine per ml were added to stop the division of glial cells.

After additional 14–16 days in culture, cells were briefly washed with PBS and fixed in methanol at −20 °C for 15 min, followed by three 10-min washing steps in buffer A (Olink Bioscience, Sweden). Permeabilization solution [0.1% triton-X100 and 10% normal donkey Serum (NDS) in buffer A], was applied to the cells for 5 min, followed by 3 washes in PBS. The primary antibodies, rabbit anti-CYP2J2 (Ma et al. 1999; kind gift from Darryl Zeldin) and goat anti-rat mEH (MEH1, Detroit R&D, USA) were diluted 1:200 in PBS containing 10% NDS, and incubated overnight at 4 °C in a humid chamber. On the next morning, coverslips were washed 3 times for 10 min in buffer A. Subsequent incubations were done in a humid chamber at 37 °C, using the Duolink in situ PLA probes and detection reagents FarRed (Olink Bioscience, Sweden). Briefly, 40 µl of the Duolink PLA probes solution (rabbit PLUS and goat MINUS), was placed on the coverslips and incubated for 1 h. After 2 washes of 5 min with buffer A, samples were incubated with the ligation solution for 1 h, followed by 2 washes of 2 min with buffer A. Samples were incubated for 2 h with the amplification solution containing detection reagent FarRed. Two final washes of 10 min were done with 1× buffer B and one wash of 1 min in 0.01× buffer B. Liquid was thoroughly tapped off the coverslips and mounted with ProLong Gold Antifade Reagent plus DAPI reagent (Molecular Probes, USA). Images were taken within 24 h.

### Statistical evaluation

Statistical analyses were performed using the software package Prism 5 for Mac OS X (www.graphpad.com). Samples to be analyzed were first subjected to a 1-way ANOVA, followed by a Dunnett’s test for the comparison of multiple data sets with a single control (Dunnett 1955).

## Results

### Construction and validation of expression vectors

YFP and CFP were chosen as the FRET pair to be N-terminally fused to the proteins of interest. To force their ER intraluminal localization, they were both equipped with an N-terminal leader peptide derived from the human ApoE protein. The ORFs for CYP and mEH were subsequently inserted in frame behind the YFP and the CFP ORF, respectively.

**Fig. 3:**
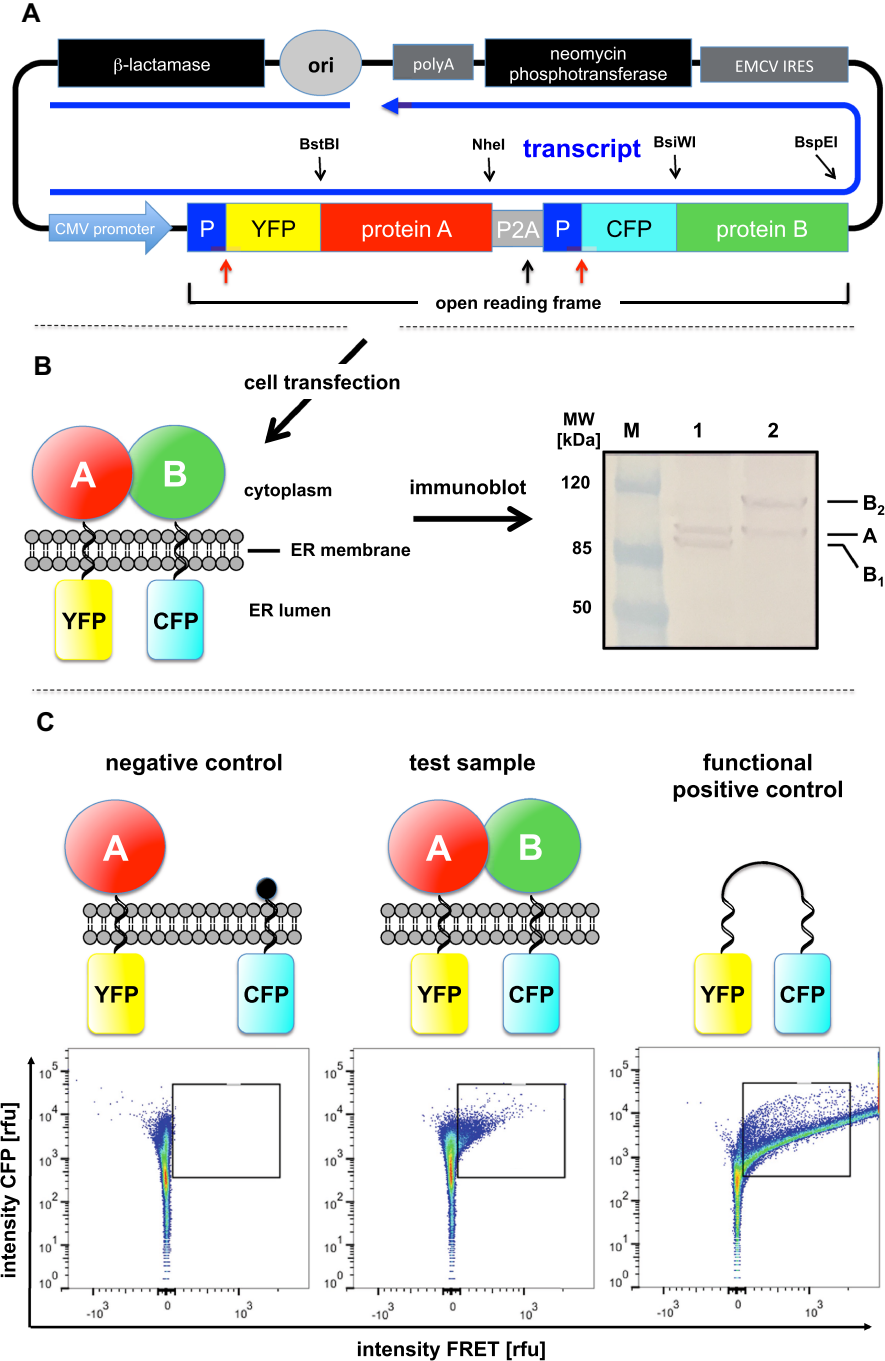
Experimental strategy for FAMPIR. **a** *Design of the expression vector.* The essential core element is an open reading frame that allows the simultaneous production of two transmembrane proteins from a single start codon, mediated by a P2A peptide sequence (gray box). The skipped peptide bond is indicated by a black arrow. Signal peptides derived from the human ApoE gene (dark blue boxes marked by “P”) direct the N-termini of the mature recombinant proteins into the ER lumen and are cleaved off during translation (cleavage sites are indicated by red arrows). Thus, the two FRET partners YFP (yellow box) and CFP (cyan box) are translocated into the lumen of the ER during protein synthesis. Their coding sequences are followed in frame by the native ORFs of the two type I transmembrane proteins (red and green boxes) to be analyzed for their interaction. Their native N-terminal signal sequences remain uncleaved and act as stop transfer signals during translation, thus resulting in a cytoplasmic orientation of the resulting proteins. The transcription of the single RNA coding for all these elements is under the control of the strong cytomegalovirus promoter (CMV; light blue box). Note that the coding sequences for the type I membrane proteins to be analyzed by FAMPIR can be conveniently exchanged using the indicated unique restriction sites. **b** *Expression products resulting after transfection of cells.* The resulting membrane topology of proteins expressed from the above construct is shown on the left. On the right, an immunoblot analysis of cell lysates obtained after transfection with two different constructs are shown. Lane 1: CYP2J5 (A) and mouse mEH (B_1_); lane 2: CYP2J5 (A) and mouse CYP reductase (B_2_). An anti-GFP antibody was used for immunostaining. **c** *FACS*-*FRET analysis.* Results of FACS-FRET analyses are displayed as diagrams with FRET intensity plotted against CFP fluorescence intensity. The protein topology of the analyzed samples is shown above each diagram. All diagrams are based on authentic data derived from the following samples: Negative control—coexpression of YFP–CYP2J5 with CFP-membrane anchor only; test sample—coexpression of YFP–CYP2J5 with CFP–mEH; functional positive control—fusion protein of YFP and CFP expressed in the cell cytoplasm

To ensure constant ratios of the two interaction partners we decided to construct bicistronic expression vectors to obtain both interaction partners from a single transcript. In a first series of experiments, we used the internal ribosomal entry side (IRES) of eIF4G that we successfully employed for similar purposes in previous work (unpublished) to initiate the synthesis of the second fusion protein coded on the above single transcript. However, transfection of HEK293 cells with such constructs reproducibly resulted in the expression of a single, unexpectedly large fusion protein that did not match in size with the proteins obtained when only YFP–CYP or CFP–mEH alone were expressed in HEK293T cells. Resequencing of the complete constructs verified the correctness of their sequences and the nature of the observed artifacts remained obscure. We, therefore, decided to replace the stop codon of the first encoded fusion protein and the IRES with a viral P2A peptide, that leads to a discontinuous polypeptide synthesis on translation, thus affording two separate proteins in stoichiometric quantities (Szymczak et al. 2004). The lack of appropriate restriction sites for the direct modification of our complex expression construct was easily overcome using the Gibson cloning protocol (Gibson et al. 2009) as described in the Supplementary Information. Transfection of HEK293 cells with the resulting expression vector (Fig. 3a) finally resulted in the expression of the two desired fusion proteins in a 1:1 ratio, as evidenced by immunoblotting using a GFP antibody for the detection of the recombinant proteins (Fig. 3b).

In the final construct, the ORFs of CYP and mEH were flanked by unique restriction sites that allowed the exchange of these ORFs against other sequences of interest (Fig. 3a). As a functional positive control for our interaction analysis, we exchanged the mEH ORF against that of the mouse CPR for which a physical interaction with CYP is essential for the catalytic activity of the latter. As a respective negative control, we chose to fuse the CFP to just a membrane anchor without a cytoplasmic protein domain being attached. We inserted the first and second helix of the rat GABA B2 receptor (Kaupmann et al. 1998; Zemoura et al. 2013) at the C-terminus of CFP, based on the reported topology of these helices. The second transmembrane helix was equipped with a C-terminal KDEL signal to ensure retention of the fusion protein in the ER (Tang et al. 1992).

### FRET analyses by confocal microscopy

Co-localization of fusion proteins in the ER of transfected cells was verified by confocal microscopy using the endoplasmic reticulum-specific dye ER-ID^®^ Red (Enzo Life Sciences, Inc.). All fusion proteins displayed the expected co-localization with the ER marker, as well as with each other in the case of the co-expressions of two fluorescent fusion proteins.

We next tried to assess the physical association of our fusion protein pairs by FRET analyses. Using a construct that allowed the cytoplasmic expression of a soluble YFP/CFP fusion protein as a technical control we established the most suitable detection procedure for our microscopy-based FRET analyses. Among a variety of different protocols, including sensitized emission and different photobleaching strategies, acceptor photobleaching gave the most sensitive and consistent results. However, none of our test constructs, including the CYP/CPR pair positive interaction control, gave a statistically significant FRET signal (Fig. S2). Because the expression yield obtained for the cytoplasmatic positive control was much higher compared to that of our ER-localized fusion proteins, we concluded that the microscopic FRET analysis—at least in our hands—was not sufficiently sensitive to pick up our apparently quite weak signals.

### FACS-based FRET analysis of membrane protein interaction in the endoplasmic reticulum (FAMPIR)

**Fig. 4:**
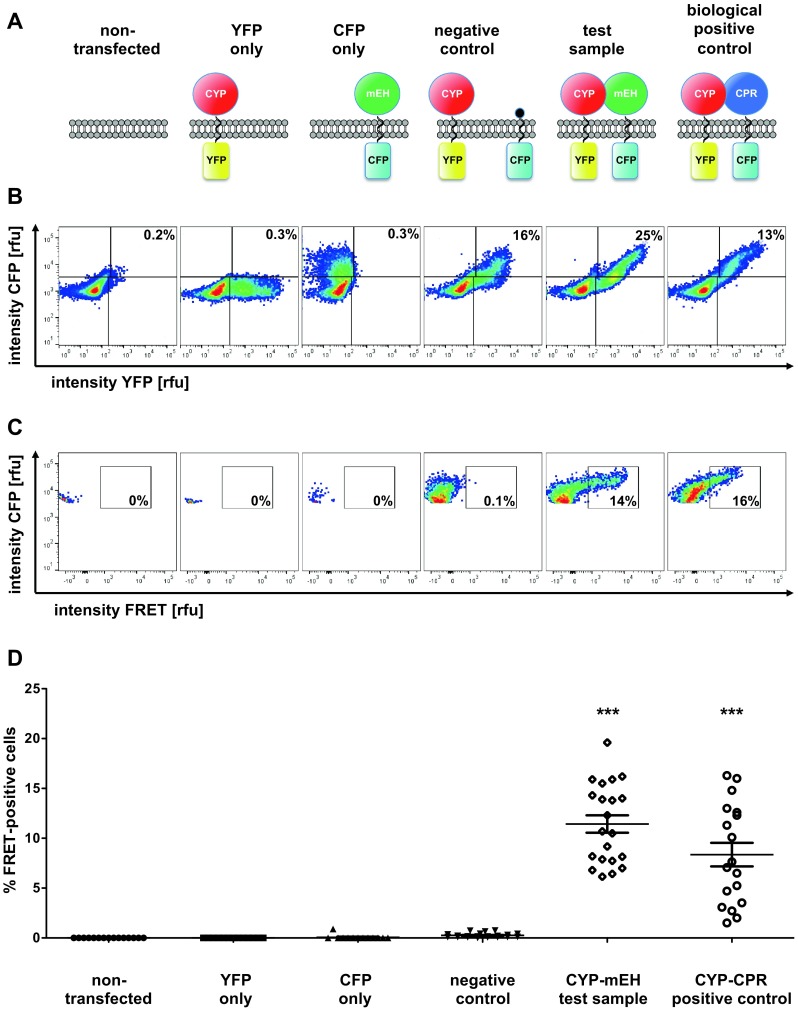
Analysis of complex formation between CYP2J5 and mouse mEH using FAMPIR. HEK 293T cells were separately transfected with 5 different constructs based on the vector represented in Fig. 3a. **a** Represents the set of recombinant proteins expressed in the respective sample. A non-transfected control was carried along as well. **b** Shows the fluorescence intensity obtained for YFP plotted against the fluorescence intensity for CFP. The horizontal and vertical line dividing the schemes into quadrants represent the thresholds finally defined for CFP (horizontal line) and YFP (vertical line) signal detection, based on the measurements obtained with the YFP only and the CFP only transfectants. Therefore, the upper right quadrant contains the fraction of cells that show detectable co-expression of the two fluorophores. The figures in the quadrants indicate the percentage of CFP/YFP-positive cells as the percentage of the whole cell population. The cells from the upper right quadrant were further analyzed for FRETing by plotting the FRET signal against the CFP fluorescence (**c**). The rectangular box inside of each panel indicates the region where cells were counted as FRET positive. The figure inside the box indicates the percentage of FRET positive cells on the basis of all CFP/YFP-positive cells. Due to the lack of CFP, YFP or both fluorophores, only very few cells of the three control samples on the left were included in this analysis. **d** Shows the cumulative results of analyses for all constructs from separate experiments (13–21 independent experiments per construct). The values obtained for the CYP2J5/CPR construct (biologic positive control) and the CYP2J5/mEH construct were compared with the CYP2J5/membrane_anchor_only construct (negative control) by a 1-way ANOVA, followed by a Dunnett’s multiple comparison test (Dunnett 1955). ***p < 0.001

To efficiently preselect for cells with sufficient fluorophore expression levels and to increase the statistical power of our analyses, we switched to a FACS-based FRET approach as recently published by Banning et al. (2010) and by Thyrock et al. (2010). Using this procedure, we could easily detect robust and reproducible FRET signals with both our CYP/CPR functional positive control as well as our test construct for the CYP/mEH interaction (Fig. 4). In contrast, our CYP/membrane_anchor_only negative control did not display any appreciable FRETing. About 10% of the cells selected for sufficient YFP and CFP expression, based on their respective fluorescence intensities, were identified as FRET positive in our test system. This percentage of cells giving a positive FRET signal did not significantly differ between the functional control and the test construct, indicating that mEH and CYP2J5 appeared to have a very similar degree of physical interaction as have CYP2J5 and CPR.

### Confirmation of the FAMPIR results with proximity ligation assay

**Fig. 5:**
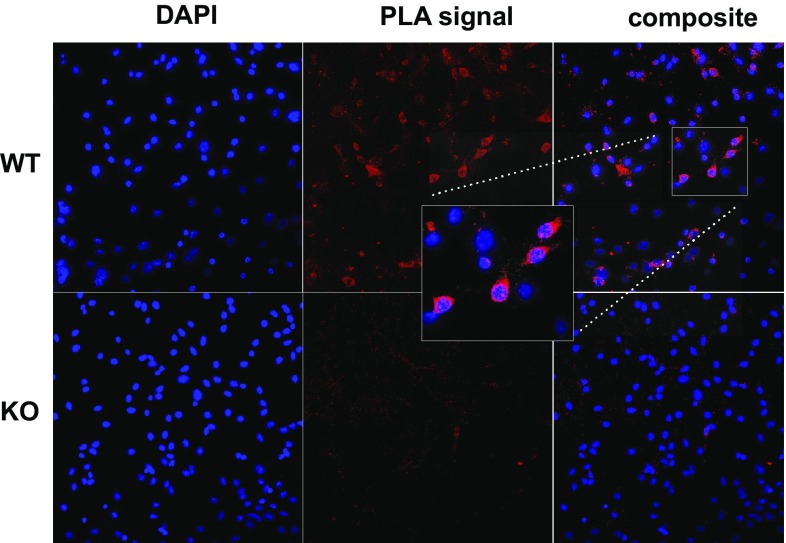
PLA analysis of the physical association of CYP2J5 and mEH in cultivated mouse hippocampal neurons. Neurons from WT and mEH−/− mouse hippocampi were isolated, cultivated and subjected to PLA analysis as described in “Methods”. The left column shows cell nuclei stained with DAPI, the middle column displays the PLA signal (red staining in case of close proximity of the two antigens tested for physical association) and the right column shows the superimposed pictures. The upper row gives the results for wild type neurons and the lower row that for mEH−/− neurons used as negative control. Additional negative controls with WT neurons where either the CYP primary antiserum or the mEH primary antiserum were also carried out but are not shown

To prove CYP2J5/mEH interaction with a second, independent method that could be used ex vivo without recombinant expression of arguably artificial fusion proteins, we employed proximity ligation assay (PLA) analyses in primary cultures from hippocampal neurons obtained from WT and mEH−/− origin. In WT neuronal cultures, a substantial fraction of the cells gave a positive PLA signal while this was essentially absent in similarly treated mEH−/− cells (Fig. 5). These results were in excellent agreement with our previous findings in recombinant HEK293 cells using FAMPIR, thereby substantiating both, the physical interaction between CYP2J5 and mEH as well as the validity of the new experimental procedure.

## Discussion

Unequivocal evidence for the specific association of membrane proteins is difficult to provide. From a theoretical consideration, at least a transient physical interaction between CYP and CPR appears essential, in view of the electron transport from CPR to CYP that initiates catalysis (Lu et al. 1969). Consequently, first models for this physical interaction have been developed very early (Peterson et al. 1976) and a plethora of studies, nicely reviewed by Kandel and Lampe (2014), have followed, including X-ray studies that give a detailed picture of plausible modes of interaction between CPR and CYP (Hamdane et al. 2009).

In contrast, the interaction of mEH and CYP is not essential for catalysis, yet an attractive possibility, in view of the potentially better control of reactive epoxide intermediates formed by CYP. Thus, the hypothesis of such an interaction has been proposed already in the early seventies by Oesch and Daly (1972). Several studies have tried to provide experimental evidence for this interaction, including analyses of enzyme kinetics (Guengerich and Davidson 1982; Oesch and Daly 1972; Taura Ki et al. 2002), affinity purification (Taura et al. 2000), cross-linking (Guengerich and Davidson 1982), and/or immunoprecipitation/immobilization (Etter et al. 1991; Fujiwara and Itoh 2014) approaches. Unfortunately, each of these approaches suffers from intrinsic limitations, leaving room for alternative explanations other than specific CYP–mEH interaction as the reason for the observed results. For instance, the early kinetic analyses were interpreted in the absence of (a) the understanding of peculiarities of the two step enzymatic mechanism of mEH that is meanwhile well described (Arand et al. 1999; Laughlin et al. 1998; Tzeng et al. 1998) and (b) the consideration of additional epoxide hydrolase isoenzymes that were identified later (Decker et al. 2012; Hammock et al. 1976). Affinity purification, immunoprecipitation and plasmon surface resonance-based procedures with purified membrane proteins share the intrinsic problem of unspecific hydrophobic interactions, due to the presence of the surface-exposed hydrophobic membrane anchors of these proteins. Finally, the crosslinking studies were unfortunately not performed with photoreactive reagents, leaving the possibility of stochastic collision events, rather than specific associations, as the basis for the observed cross-linking.

Thus, there was still the need for the unequivocal assessment of mEH–CYP interaction and a non-invasive detection technology seemed the most suitable for such analyses. We, therefore, developed the present FRET-based FAMPIR approach.

FRET can occur between YFP and CFP, our chosen pair of fluorophores, if the distance between the two is below 10 nm, which is close to the approximate diameter of CYP or mEH (approx. 7 nm). We reasoned that attaching the FRET partners YFP and CFP to the short intra-ER-luminal N-termini of CYP and mEH would most likely result in a permissive distance (or the least steric hindrance) for FRET interactions if the catalytic subunits of mEH and CYP would associate on the other side of the membrane. To ensure the ER luminal localization of YFP and CFP, their coding regions were inserted downstream and in frame of a sequence coding for the human ApoE signal peptide. The C-terminus of the fluorescent proteins was fused to the N-termini of the membrane proteins to be tested in the interaction analysis, thus providing their native membrane topology. Use of the P2A peptide (Szymczak et al. 2004) to fuse the reading frames of the two FRET partners resulted in the synthesis of both fusion proteins in an approximately 1:1 ratio (Fig. 3b), favorable for a sensitive FRET analysis. As a “native” positive control, based on the reasoning provided above, we used CYP2J5 and CPR fusion proteins as FRET pair. As a negative control, we combined the CYP2J5 fusion protein with an artificial construct composed of a CFP fusion to the two N-terminal transmembrane helices of the GABA B2 receptor, followed by a KDEL sequence. Although KDEL was originally described as an ER retention signal for soluble ER intraluminal proteins, its efficacy to retain integral plasma membrane proteins in the ER has been experimentally demonstrated by different groups (Bailey et al. 2013; Tang et al. 1992).

Transfection of the respective constructs provided detectable ER-resident expression of the respective fluorescent fusion proteins. However, attempts to microscopically detect FRETting, even with the CYP/CPR positive control, were unsuccessful. We therefore included a direct fusion of YFP and CFP, expressed soluble in the cytoplasm of transfected cell, as a functional positive control to assess our methodologic capability to detect FRET, in principle (Fig. 3c, Fig. S2). This construct resulted in reproducible detection of FRETting using acceptor photobleaching, the most sensitive of the different methods we employed for microscopic FRET analyses. An obvious difference between this positive control and our test samples was the expression yield of fluorescent proteins that differed by about an order of magnitude, in favor of the soluble fusion protein. We reasoned that, in combination with a potentially higher FRET efficiency of this fusion protein compared to our test pair, it might be the sensitivity of the recording system that precludes the identification of a positive FRET signal. We, therefore, switched to the recently published FACS-FRET analysis (Banning et al. 2010; Thyrock et al. 2010), as described above, and indeed obtained statistically significant results indicating the interrogated association between CYP2J5 and mEH (Fig. 4d). The fact that only a small fraction of the cells in the analyses using either CYP/mEH or CYP/CPR (only around 5–20% FRET-positives in the already preselected subpopulation with sufficient YFP and CFP expression) gave a positive signal is very well in line with our failure to detect this FRET signal by microscopic analysis, where only a limited number of cells can be analyzed per session. This obvious sensitivity issue can be nicely overcome by the strength of the FACS analysis, i.e., the rapid scanning of many thousand cells in a short period of time. It is striking to see that the CYP/mEH test pair behaves very similar to the CYP/CPR positive control, suggesting a qualitatively and quantitatively quite similar mode of association of CYP2J5 with these two partners. The PLA assay, which used an in situ approach to assess the mEH–CYP interaction using primary cultures of hippocampal neurons, further corroborated the FACS–FRET findings and, on top, provided evidence for the physical association of native CYP2J and mEH in non-transgenic cells.

In conclusion, our present results clearly demonstrate the physical association between CYP2J5 and mouse mEH. Future analyses will show how far this interaction with mEH extends to other CYP isoforms, in particular those that are involved in the formation of reactive epoxide intermediates from xenobiotics, to allow the immediate processing of these otherwise potentially carcinogenic metabolites. CYP2J5 itself has been characterized as an arachidonic acid epoxygenase (Ma et al. 1999). The colocalization of CYP2J5 and mEH in mouse hippocampal neurons (Figs. 2, 5), together with our previous finding that mEH contributes to the cerebral turnover of arachidonic acid epoxides (EETs; Marowsky et al. 2009), suggests an important role of mEH in the regulation of these important endogenous signaling molecules. In line with this, we recently found that mEH has a significant effect on blood perfusion in the mouse brain (Marowsky et al. 2016). However, this is best explained by the expression of mEH in the cerebral vascular endothelium (Marowsky et al. 2009) where it is ideally situated to process vasodilatory EETs.

## Electronic supplementary material

Below is the link to the electronic supplementary material.

Supplementary material 1 (PDF 3365 kb)
