# Supplementary material for: Evidence for a complex formation between CYP2J5 and mEH in living cells by FRET analysis of membrane protein interaction in the endoplasmic reticulum (FAMPIR)
Source: Arch Toxicol. 2017 Oct 13;91(11):3561–70. doi: 10.1007/s00204-017-2072-0 (PMC5696493; doi:10.1007/s00204-017-2072-0)
Supplement: Supplementary file 1 — Supplementary material 1 (PDF 3365 kb) [file 204_2017_2072_MOESM1_ESM.pdf]

Supplementary information

Vector construction

The expression constructs were based on the eukaryotic expression vector pIRES neo (Clontech). Important features of this plasmid are (i) a CMV promoter to drive transgene expression in a large selection of eukaryotic cells, (ii) the polylinker downstream of the promoter to facilitate incorporation of foreign DNA, followed by (iii) a neomycin phosphotransferase gene preceded by an IRES sequence to allow for the translation of this aminoglycoside resistance-conferring enzyme from the same mRNA that also codes for the transgenic protein. The latter allows for the selection of high transgene expressing cells after stable transfection, a procedure actually not employed in the present study. First, the polylinker of pIRESneo was modified by the introduction of 4 new unique restriction sites for the enzymes BstBI, NheI, BsiWI and BspEI, to allow the comfortable exchange of target protein coding sequences in the final construct (Fig. S1 ①). This was achieved by incorporating a synthetic double stranded oligonucleotide (sense: 5'-GGCCGCTTCGAAGCGCTAGCCGGCGTACGCGTCCGG-3'; antisense: 5'-AATTCCGGACGCGTACGCCGGCTAGCGCTTCGAAGC-3') into the NotI/EcoRI-restricted pIRESneo. Next, the 300 bp IRES of the eukaryotic translation initiation factor 4 gamma 1 (eIF4G1) was amplified from human liver cDNA using the primers 5'-GAAGGCTAGCCCGGGGTAGGGATGAGGGAGGGA-3' and 5'-GAAGCGTACGTCGACTGATATCCTTTCCTCCTTGG-3' with Pfu DNA polymerase and subsequently cloned into the NheI/BsiWI site of the above construct (Fig. S1 ②). In order to afford ER intraluminal expression of YFP and CFP, their respective cDNAs were fused to oligonucleotides coding for the N-terminal leader peptide of the human ApoE protein. Thus, the YFP gene of pcDNA6.2-N-YFP.DEST (Thermo Scientific) was amplified using the oligonucleotides 5'-CAACGGTACCATCGATCGCCGCC*ATGAAGGTTCTGTGGGCTGCGTTGCTGGTCACATTCCTGGCAGGATGCCAGGCCAAGGTGGAGCAAGCGGTG*AGCAAGGGCGAGGAGCTGTTCACC-3' (the sequence coding for the ApoE leader peptide is given in italics) and 5'-CAACTTCGAACTTGTACAGCTCGTCCATGCCGAGAG-3' as forward and reverse primer, respectively. After restriction with ClaI and BstBI, the resulting amplicon was ligated into the BstBI restriction site of the vector containing the eIF4G1 IRES (Fig. S1 ③). Using the same forward primer and the oligonucleotide 5'-CAACCGTACGCTTGTACAGCTCGTCCATGCCGAGAG-3' as the reverse primer, the CFP open reading frame from the plasmid prSET-CFP (Invitrogen) was amplified and, after restriction with Aco65I and BsiWI, inserted into the vector resulting from the above described YFP incorporation (Fig. S1 ④). Next, the CYP2J5 cDNA was amplified from C57/BL6 mouse kidney cDNA using the primers 5'-CAAGTTCGAAATGATTATGTTTTTGAGCTCCC-3' (CYP2J5 forward) and 5'-ATTAGCTAGCTCACACTCTAGGGATAGCACAAA-3' (CYP2J5 reverse), and cloned into the BstBI/NheI site of the expression construct to yield an in-frame fusion of the CYP2J5 open reading frame with that of the preceding ApoE/YFP protein (Fig. S1 ⑤). To obtain the final expression constructs, one of the following three cDNAs was inserted into the BsiWI/BspEI restriction sites, resulting in the plasmids for coexpression of CYP2J5 and a membrane anchor only (Fig. S1 ⑥), CYP2J5 and CPR (Fig. S1 ⑦), or CYP2J5 and mEH (Fig. S1 ⑧), as fusion proteins suitable for FRET analysis. To create a membrane anchor providing the correct

topology but being certainly unrelated to the CYP system, a short segment of the rat GABA B2 receptor coding for transmembrane helices 1 and 2 was amplified from a GABA B2 expression plasmid (Zemoura et al. 2013) using the primers 5'-TACGTACGTATAGCATCCTGTCCGCTCTCACCA-3' (GABA anchor forward) and 5'-GTTCCGGACTACAGTTCATCCTTGGTCTTTTCTGAGACGAAGGACCCA-3' (GABA anchor reverse). Mouse CPR cDNA was amplified from the above kidney cDNA using the primers 5'-ATTACGTACGATGGGGGACTCTCACGAAGACACCAGT-3' (CPR forward) and 5'-GAAGTCCGGACTAGCTCCATACATCCAGCGAGTA-3' (CPR reverse). Mouse mEH cDNA was amplified from the plasmid pET20mmEH, (Marowsky et al. 2009) using the primer pair 5'-ATTACGTACGATGTGGCTGGAACTCATCC-3' (mEH forward) and 5'-ATTATCCGGATCACTGCAGCTCAGCCAGGG-3' (mEH reverse). For the proper adjustment of the FRET recording conditions, constructs that mediate the expression of either a CFP fusion protein only or a YFP fusion protein only were required. Therefore, the above construct harboring the CYP2J5 and the mEH cDNAs was cut with NheI and BspEI and re-ligated after filling in of the sticky ends to obtain pYFP_CYP2J5 (Fig. S1 ⑨). Using the same starting construct, restriction with EcoRV and direct re-ligation led to the expression vector pCFP_mEH (Fig. S1 ⑩).

Because the co-expression of the desired fusion proteins mediated by the eIF4G1 IRES did not lead to success (see Results), the CYP2J5 stop codon and the subsequent IRES were replaced by a P2A sequence to allow simultaneous translation of both fusion proteins from the same vector in a single go, taking advantage from the skipping of one particular peptide bond formation within the P2A sequence to afford production of two separate proteins (Szymczak et al. 2004). This was achieved by the modification of the CYP2J5/membrane_anchor_only construct (see above), employing the Gibson cloning protocol (Gibson et al. 2009) to compensate for the lack of suitable restriction sites. Two fragments were amplified from the construct CYP2J5/membrane_anchor_only using the primer pairs 5'-GGGATCACTCTCGGCATGGACGA-3' (YFP_CYP forward) and 5'-GGGTCCAGGATTCTCCTCGACGTCACCAGCCTGCTTAAGCAGGCTGAAGTTGGTGGCTCCACTTCCGCTAGCCACTCTAG GGATAGCACAAA-3' (CYP_P2A reverse; Fig. S1 ⑪), and 5'-CTTAAGCAGGCTGGTGACGTCGAGGAGAATCCTGGACCCGATATCGATAGCGCCGCCATGAAGGTTCTGTGG-3' (P2A_anchor forward) and 5'-AATTAATTCCAGCACACTGGCGG-3' (anchor_stop reverse; Fig. S1 ⑫) to yield fragments of 1.6 and 1.1 kbp, respectively, that shared a 39 bp overlap comprising the synthetic P2A sequence. The construct CYP2J5/membrane_anchor_only was digested with BstBI and BamHI to yield a large 5 kbp fragment with 35 - 40 bp overlap to the outer ends of the above amplified sequences (Fig. S1⑬). The three fragments were fused to each other in a one-pot reaction according to the procedure described by Gibson and colleagues (Fig. S1 ⑭). In the resulting construct pCYP2J5_P2A_anchor, the BsiWI/BspEI membrane anchor fragment was subsequently replaced by either the corresponding mouse mEH cDNA fragment (Fig. S1⑮) or the CPR cDNA fragment (Fig. S1⑯) to yield pCYP2J5_P2A_mEH and pCYP2J5_P2A_CPR.

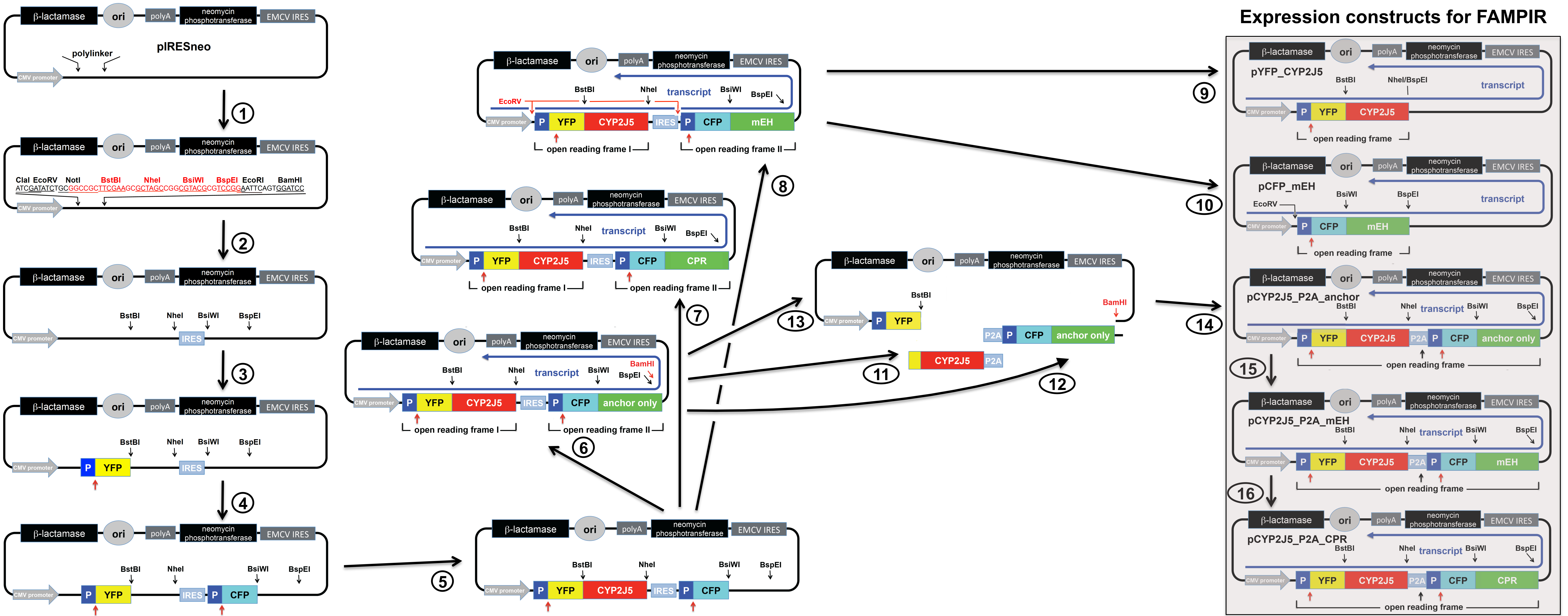

Expression constructs for FAMPIR
pIRESneo
polylinker
β-lactamase
ori
polyA
neomycin phosphotransferase
EMCV IRES
CMV promoter
ClaI EcoRV NotI BstBI NheI BsiWI BspEI EcoRI BamHI
ATCGATATCTGCGGCCGCTTCGAAGCGCTAGCCGGCGTACGCGTCCGGAATTCAGTGGATCC
IRES
P
YFP
CFP
CYP2J5
mEH
CPR
anchor only
P2A
transcript
open reading frame I
open reading frame II
open reading frame
pYFP_CYP2J5
NheI/BspEI
pCFP_mEH
pCYP2J5_P2A_anchor
pCYP2J5_P2A_mEH
pCYP2J5_P2A_CPR
1
2
3
4
5
6
7
8
9
10
11
12
13
14
15
16

Fig. S1 Construction of the expression vectors for the FRET analyses

1. Modification of the multiple cloning site of the starting vector pIRESneo to insert unique sites for BstBI, NheI, BsiWI and BspEI; 2. Insertion of the second IRES into the NheI/BsiWI site; 3. Insertion of the YFP cDNA preceded by the ApoE signal peptide (P) into the BstBI site; 4. Insertion of the CFP cDNA preceded by the ApoE signal peptide (P) into the BsiWI site; 5. Insertion of the CYP2J5 cDNA into the BstBI/NheI site; 6. Insertion of the GABA anchor cDNA into the BsiWI/BspEI site; 7. Insertion of the CPR cDNA into the BsiWI/BspEI site; 8. Insertion of the mEH cDNA into the BsiWI/BspEI site; 9. Elimination of the NheI/BspEI fragment; 10. Elimination of the EcoRV fragment; 11. PCR amplification of a stop codon-free CYP2J5 cDNA with a 5' fusion to YFP and a 3'fusion to P2A; 12. PCR amplification of the signal peptide/CFP/GABA anchor cDNA with a 5' fusion to P2A and a 3' non-translating vector sequence extending 35 bp beyond the BamHI restriction site; 13. Isolation of the large BstBI/BamHI fragment; 14. Gibson cloning of the three fragments generated in steps 11 – 13; 15. Replacement of the BsiWI/BspEI fragment by the mEH cDNA; 16. Replacement of the BsiWI/BspEI fragment by the CPR cDNA.

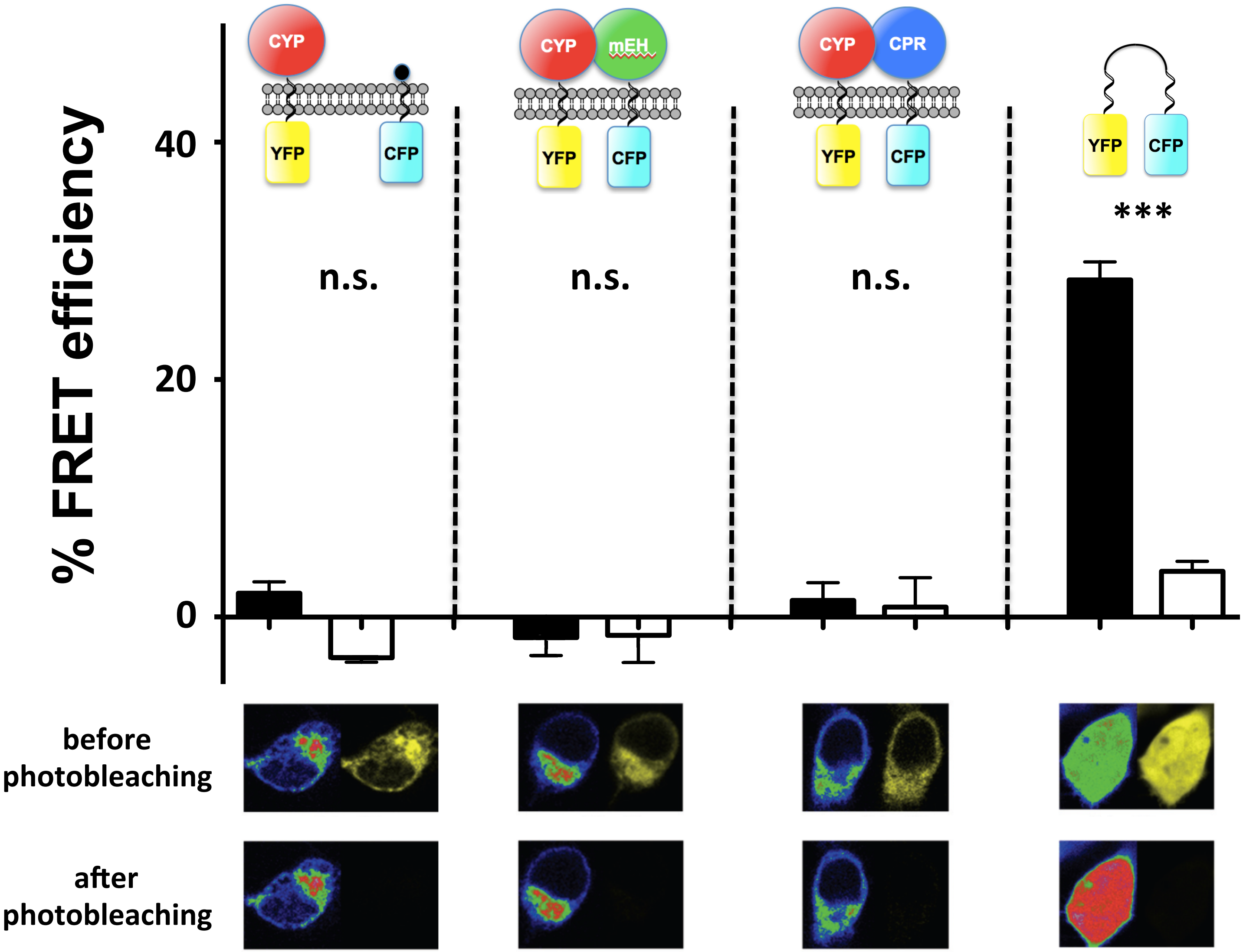

% FRET efficiency
40
20
0
CYP
YFP
CFP
CYP
mEH
YFP
CFP
CYP
CPR
YFP
CFP
YFP
CFP
n.s.
n.s.
n.s.
***
before photobleaching
after photobleaching

Fig. S2 Results of the microscopic FRET analyses using acceptor photobleaching
FRET analysis by acceptor photobleaching was performed as described in Materials and Methods. CYP2J5/membrane_anchor_only (negative control), CYP2J5/mEH (test sample), CYP2J5/CYP reductase (biological positive control) and soluble YFP/CFP fusion protein (technical positive control) pairs were tested for FRET efficacy as shown in the bar diagram in the above order from left to right. Black bars show the results after photobleaching. As a negative internal control, two consecutive measurements of the same sample were taken without intermittent photobleaching (white bars). The FRET efficiency was calculated according to the following formula:

$$\% \text{ FRET efficiency} = (I_{CFPa} - I_{CFPb})/I_{CFPa} \times 100$$

I_{CFPb} is the CFP fluorescence intensity before and I_{CFPa} is the CFP fluorescence intensity after photobleaching. Each bar represents the average of 2 to 18 separate measurements. Individual t-tests were run with each experimental set-up comparing the results after photobleaching (black bars) with that obtained for the negative controls (white bars). After Bonferroni correction, a value for $p < 0.0125$ was considered statistically significant. n.s. = not significant; *** = $p < 0.0001$. Underneath each pair of bars, a typical pair of pictures for an analyzed cell is shown before and after YFP photobleaching (CFP fluorescence on the left, YFP fluorescence on the right).
